# Association between Changes in Stent Graft Volume and Type II Endoleaks after Endovascular Aneurysm Repair

**DOI:** 10.3400/avd.oa.25-00136

**Published:** 2026-03-11

**Authors:** Satoshi Yamamoto, Takuya Hashimoto, Takashi Endo, Masaya Sano, Osamu Sato, Juno Deguchi

**Affiliations:** 1 Department of Vascular Surgery, Saitama Medical Center, Saitama Medical University, Kawagoe, Saitama, Japan; 2 Department of Cardiovascular Surgery, Ome Medical Center, Ome, Tokyo, Japan

**Keywords:** endovascular aneurysm repair, Type II endoleak, stent graft volume

## Abstract

**Objectives:**

Type II endoleaks (T2ELs) after endovascular aneurysm repair (EVAR) are associated with delayed adverse events. This study evaluated the association between changes in stent graft volume (SgV) and T2EL after EVAR.

**Methods:**

This study included 127 patients who underwent EVAR for abdominal aortic aneurysms between 2010 and 2022. SgVs were calculated using computed tomography, and SgV ratios were calculated by dividing SgV 2 years after EVAR by SgV immediately after EVAR. The association between the SgV ratio and T2EL 2 years after EVAR was evaluated.

**Results:**

T2ELs were detected in 62 patients (49%) 2 years after EVAR. Univariate analysis revealed that age, number of patent lumbar arteries, expanded polytetrafluoroethylene (ePTFE) stent grafts, and the SgV ratio were significantly correlated with T2EL. Multivariate analysis showed that age ≥80 years, patent lumbar arteries ≥4, ePTFE stent graft, and SgV ratio ≤1.23 were independently associated with T2EL. In both the ePTFE (80 patients) and polyester (47 patients) stent graft groups, the SgV ratio was independently associated with T2EL.

**Conclusions:**

The SgV ratio was significantly correlated with T2EL and may improve T2EL surveillance after EVAR.

## Introduction

Endovascular aneurysm repair (EVAR) has been established as the treatment for abdominal aortic aneurysms (AAA) owing to its low mortality and complication rates. However, endoleaks can lead to EVAR failure. Type II endoleaks (T2ELs) after EVAR are the most prevalent type of endoleak.^1)^ The data of 17099 patients from the registry of the Japanese Committee for Stentgraft Management showed a correlation between persistent T2EL and delayed adverse events, including mortality after EVAR due to aneurysm sac enlargement, reintervention, rupture, and abdominal aortic aneurysm.^2)^ Persistent T2EL is a serious issue in radical treatment for AAA.^1,3,4)^ However, it is difficult to continue monitoring endoleaks using contrast radiography over the entire period after EVAR. T2EL often resolves spontaneously, although it is sometimes newly detected during follow-up.^2,5,6)^ Doppler ultrasound has poor sensitivity for endoleak detection, and contrast-enhanced computed tomography (CECT) is considered the standard for endoleak surveillance.^7)^ However, repeated CECT could lead to contrast-media-induced renal dysfunction and allergy compared with non-contrast CT. Trans-arterial angiography is invasive and is not routinely performed. Clinically, plain CT is often used only to check the aneurysm sac size. Therefore, an alternative indicator of T2EL is required.

We have observed that the degree of change in stent graft volume (SgV) differs among patients, although all stent grafts increase in volume over time compared to immediately after EVAR. Specifically, stent grafts show attenuated expansion when aneurysm sacs enlarge drastically because of endoleaks. We hypothesized that the degree of SgV change is associated with T2EL and may indicate EVAR success. Therefore, in this study, we investigated the association between changes in SgV and T2EL after EVAR.

## Materials and Methods

### Data collection

**Fig. 1:**
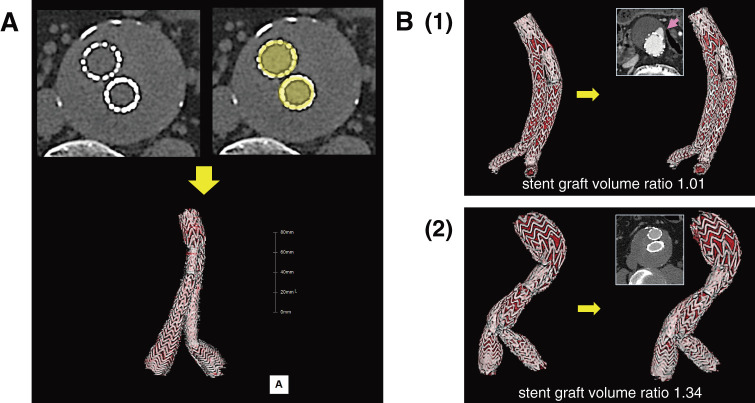
Stent graft volume measurement. (**A**) Axial plain CT images before and after tracing the stent border lines, and a 3-dimensional image reconstructed from plain CT images. Stent graft volume is calculated as the integrated value by tracing the outer stent border from top to bottom in the axial images of plain CT. The inner area of the stent is displayed in red if a 3-dimensional image is reconstructed from plain CT images, and the red area in the 3-dimensional image is not contrasted. (**B**) Images of 2 cases as examples: (**1**) the stent graft with Type II endoleaks (T2EL) *(arrow)* and (**2**) the stent graft without T2EL. The 3-dimensional image of the stent graft within 1 month after EVAR (left) and the axial CT and 3-dimensional images of the stent grafts 2 years after EVAR (right) are shown for each case. The axial contrast-enhanced CT images clarify the existence or absence of T2EL, but stent graft volume is measured on plain CT images. The SgVs were 54.5 mL initially and 55.0 mL 2 years after EVAR, and the SgV ratio was calculated as 1.01 in Case 1. The SgVs were 83.8 mL initially and 111.9 mL 2 years after EVAR, and the SgV ratio was calculated as 1.34 in Case 2. EVAR: endovascular aneurysm repair; ePTFE: expanded polytetrafluoroethylene; CT: computed tomography; SgVs: stent graft volumes

Between 2010 and 2022, 127 patients at Saitama and Ome Medical Centers who underwent EVAR for AAA and CT for the first time within 1 month and 2 years after EVAR were included in this study. Patients treated with the AFX (Endologix, Irvine, CA, USA) stent graft, which had an inner metallic endoskeleton, were excluded. None of the patients had Type I and/or Type III endoleaks at surgery. Values, including SgV, were measured on the initial and 2-year plain CT images. SgV was approximated by tracing the stent border from the top to the bottom of the stent graft in the axial images using Ziostation 2 (Ziosoft, Tokyo, Japan) (**Fig. 1**). SgVs were measured twice by physicians. In the Endurant (Medtronic, Minneapolis, MN, USA) and Zenith (Cook Medical, Bloomington, IN, USA) stent grafts, the non-fabric-covered top stents were not measured. In the Aorfix (Lombard Medical, Oxfordshire, UK) stent graft, the measurement boundary was defined as the midpoint of the fish-mouth-shaped portion’s ends. A 3-dimensional image was reconstructed in parallel using these procedures. The SgV ratio was calculated by dividing SgV at 2 years after EVAR by SgV immediately after EVAR. The overall length of the stent graft (SgL) was measured as the sum of the lengths of the main trunk portion, right leg, and left leg. The SgL ratio was calculated by dividing SgL at 2 years after EVAR by SgL immediately after EVAR. T2EL was assessed in CECT images or angiography, referring to clinical information, including Doppler ultrasound data. In this study, any contrast enhancement in the aneurysm sac or around the inferior mesenteric and lumbar arteries was considered T2EL. Cases with Type I, III, or IV endoleaks 2 years after EVAR were excluded.

### Endpoints

Patients with T2EL 2 years after EVAR were considered the discriminant groups. Retrospectively, clinical findings and calculated data, including the SgV ratio, were compared between patients with T2EL and those without it, 2 years after EVAR.

### Statistical analysis

Data are presented as means and standard deviations (SDs). Student’s t-test or chi-square/Fisher’s exact test was used for statistical analysis. The Youden index and the receiver operating characteristic curve were used to calculate the cut-off values of the SgV ratio, number of patent lumbar arteries, and diameter of the inferior mesenteric artery. The threshold for statistical significance was set at *p* <0.05. Multivariate analyses were performed to identify the risk factors for T2EL 2 years after EVAR. Only factors that achieved a level of *p* <0.05 after univariate testing were entered in the logistic regression analysis. Similarly, outcomes were evaluated, and values, including the SgV ratio, were compared in the expanded polytetrafluoroethylene (ePTFE) stent grafts and in the polyester stent grafts, respectively.

This study followed the ethical guidelines for medical research involving human subjects and was approved by the Institutional Review Board of Saitama Medical Center (approval no. 2024-142).

## Results

### Patient characteristics and outcomes

Patient characteristics and outcomes are presented in **Table 1**. In the whole cohort, SgV was 64.5 ± 16.4 mL at the first time point and 76.7 ± 21.5 mL at 2 years after EVAR, yielding an SgV ratio of 1.19 ± 0.12. Endoleaks were detected in 62 (49%) patients 2 years after EVAR, and all endoleaks were categorized as Type II. They resolved spontaneously in 8 patients and occurred de novo in 10 patients during the 2-year follow-up. Among the 62 T2ELs, 15 were from the inferior mesenteric and lumbar arteries, 6 from the inferior mesenteric artery, and 41 from the lumbar arteries.

**Table 1:** Patient background and outcomes

Variables	Total (n = 127)	Type II endoleak 2 years after EVAR		*p*-value
		(+) (n = 62)	(−) (n = 65)	
Preoperative factors				
Age, years	76.6 ± 6.9	78.4 ± 7.0	75.0 ± 6.5	0.0058
Male	113 (89%)	54 (87%)	59 (91%)	0.58
Comorbid diseases				
Hypertension	104 (82%)	52 (84%)	52 (80%)	0.65
Dyslipidemia	72 (57%)	33 (53%)	39 (60%)	0.48
Diabetes mellitus	32 (25%)	15 (24%)	17 (26%)	0.84
Coronary artery disease	55 (43%)	25 (40%)	30 (46%)	0.59
Chronic heart failure	7 (6%)	5 (8%)	2 (3%)	0.27
Cerebrovascular disease	21 (17%)	9 (15%)	12 (18%)	0.64
Chronic renal failure, Grade 4 and 5	14 (11%)	4 (6%)	10 (15%)	0.16
Grade 1, 2	54	26	28	
Grade 3a	40	21	19	
Grade 3b	19	11	8	
Grade 4	9	2	7	
Grade 5	5	2	3	
Respiratory disorder	32 (25%)	16 (26%)	16 (25%)	0.84
Medications				
Antiplatelet and/or anticoagulant drug	71 (56%)	35 (56%)	36 (55%)	1.0
Antiplatelet drug use	61 (48%)	30 (48%)	31 (48%)	1.0
Anticoagulant drug use	16 (13%)	7 (11%)	9 (14%)	0.79
Statin use	61 (48%)	31 (50%)	30 (46%)	0.72
β blocker use	25 (20%)	15 (24%)	10 (15%)	0.27
Anatomic features				
Saccular	21 (17%)	11 (18%)	10 (15%)	0.81
Comorbid common iliac artery aneurysm	53 (42%)	30 (48%)	23 (35%)	0.15
Comorbid thoracic aortic aneurysm	30 (24%)	10 (16%)	20 (31%)	0.062
Sac diameter at EVAR, mm	54.3 ± 9.4	53.0 ± 6.8	55.6 ± 11.3	0.11
Proximal neck diameter, mm	24.4 ± 3.6	24.0 ± 3.3	24.7 ± 3.9	0.29
Proximal neck length, mm	32.3 ± 11.3	33.2 ± 1.0	31.5 ± 12.4	0.40
Patent inferior mesenteric artery	102 (80%)	50 (81%)	52 (80%)	1.0
Inferior mesenteric artery diameter, mm	3.1 ± 0.8	3.2 ± 0.7	3.0 ± 0.8	0.16
Number of patent lumbar arteries	3.8 ± 1.6	4.4 ± 1.4	3.2 ± 1.6	<0.0001
Stent graft				
Stent grafts made of expanded polytetrafluoroethylene	80 (63%)	54 (87%)	26 (40%)	<0.0001
Excluder	80	54	26	
Stent grafts made of polyester	47 (37%)	8 (13%)	39 (60%)	
Endurant	29	4	25	
Zenith	16	3	13	
Aorfix	2	1	1	
Postoperative details				
Stent graft volume				
Stent graft volume within 1 month after EVAR, ml	64.5 ± 16.4 (range 25.1–112.4)	62.9 ± 14.9 (range 35.0–112.4)	66.0 ± 17.7 (range 25.1–109.9)	0.28
Stent graft volume 2 years after EVAR, ml	76.7 ± 21.5 (range 27.0–139.7)	71.4 ± 17.9 (range 37.9–133.5)	81.7 ± 23.5 (range 27.0–139.7)	0.0061
Stent graft volume ratio 2 years after EVAR	1.19 ± 0.12 (range 1.002–1.569)	1.14 ± 0.08 (range 1.002–1.369)	1.23 ± 0.13 (range 1.021–1.569)	<0.0001
Stent graft volume ratio 2 years after EVAR ≤1.23	–	56 (90%)	31 (48%)	<0.0001
Stent graft length				
Stent graft length within 1 month after EVAR, mm	294 ± 56	299 ± 50	289 ± 62	0.35
Stent graft length 2 years after EVAR, mm	296 ± 58	302 ± 52	291 ± 63	0.28
Stent graft length ratio 2 years after EVAR	1.007 ± 0.022 (range 0.927–1.067)	1.010 ± 0.020 (range 0.968–1.054)	1.005 ± 0.023 (range 0.927–1.067)	0.17
Sac diameter				
Sac diameter 2 years after EVAR, mm	50.3 ± 11.7	53.0 ± 9.4	47.8 ± 13.0	0.011

Data are presented as number (%), mean ± standard deviation, or range.

EVAR: endovascular aneurysm repair

### Comparison of patients with T2EL and those without, 2 years after EVAR

#### All cases

A comparison of patients with T2EL and those without 2 years after EVAR is presented in **Table 1**. SgV was smaller in patients with T2EL than in those without, 2 years after EVAR (*p* = 0.0061), although there was no significant difference in SgV between the 2 groups at EVAR (*p* = 0.28). The SgV ratio was smaller in patients with T2EL than in those without, 2 years after EVAR (*p* <0.0001). There was no significant difference in sac diameter between the 2 groups at the time of EVAR (*p* = 0.11). However, sac diameter was larger in patients with T2EL than in those without, 2 years after EVAR (*p* = 0.011). The correlation coefficient between sac diameter change and the SgV ratio was −0.28, suggesting a weak negative correlation. The correlation coefficient between SgL and the SgV ratio was 0.067. Univariate analysis revealed that age, number of patent lumbar arteries, ePTFE stent grafts, and SgV ratio were significantly correlated with T2EL. An SgV ratio value of 1.23 was calculated as the cut-off (positive predictive value; 64%, negative predictive value; 85%, sensitivity; 52%, specificity; 90%, and area under the curve [AUC]; 0.74). Regarding the number of patent lumbar arteries, a value of 4 was calculated as the cut-off (sensitivity 58%, specificity 79%, AUC 0.70). Multivariate analysis revealed that factors independently associated with T2EL in all cases were the number of patent lumbar arteries ≥4, ePTFE stent grafts, and SgV ratio ≤1.23 (**Table 2**).

**Table 2:** Multivariate analysis of Type II endoleaks, 2 years after EVAR (logistic regression model) (all cases)

ALL cases (n = 127)		
Variable	OR (95% CI)	*p*-value
Age ≥80 years	2.89 (0.90–6.79)	0.072
Number of patent lumbar arteries ≥4	4.24 (1.65–10.91)	0.0020
Stent grafts made of expanded polytetrafluoroethylene	5.55 (1.96–15.68)	0.0008
Stent graft volume ratio ≤1.23	7.16 (2.22–23.11)	0.0005

OR: odds ratio; CI: confidence interval; EVAR: endovascular aneurysm repair

#### ePTFE stent graft subgroup

Univariate analysis revealed that age, number of patent lumbar arteries, and the SgV ratio were significantly correlated with T2EL (**Table 3**). An SgV ratio value of 1.21 was calculated as the cut-off (positive predictive value 74%, negative predictive value 56%, and sensitivity 38%, specificity 85%, AUC 0.59). Concerning the number of patent lumbar arteries, the cut-off was calculated as 4 (sensitivity 54%, specificity 78%, AUC 0.67). Multivariate analysis revealed that age ≥80 years and SgV ratio ≤1.21 were independently associated with T2EL (**Table 4**).

**Table 3:** Comparison of patients with Type II endoleaks and those without, 2 years after EVAR (expanded polytetrafluoroethylene and polyester stent grafts)

Variable	Expanded polytetrafluoroethylene stent grafts			Polyester stent grafts		
	Type II endoleak 2 years after EVAR		*p*-value	Type II endoleak 2 years after EVAR		*p*-value
	(+) (n = 54)	(−) (n = 26)		(+) (n = 8)	(−) (n = 39)	
Preoperative factors						
Age	78.6 ± 7.0	74.4 ± 5.8	0.0072	77.0 ± 7.0	75.4 ± 7.0	0.57
Male	48 (89%)	26 (100%)	0.17	6 (75%)	33 (85%)	0.61
Comorbid diseases						
Hypertension	46 (85%)	22 (85%)	1.0	6 (75%)	30 (77%)	1.0
Dyslipidemia	29 (54%)	14 (54%)	1.0	4 (50%)	25 (64%)	0.69
Diabetes mellitus,	15 (28%)	4 (15%)	0.27	0 (0%)	13 (33%)	0.086
Coronary artery disease	24 (44%)	9 (35%)	0.47	1 (13%)	21 (54%)	0.052
Chronic heart failure	5 (9%)	1 (4%)	0.66	0 (0%)	1 (3%)	1.0
Cerebrovascular disease	6 (11%)	4 (15%)	0.72	3 (38%)	8 (21%)	0.37
Chronic renal failure, Grade 4 and 5	3 (6%)	2 (8%)	1.0	1 (13%)	8 (21%)	1.0
Respiratory disorder	15 (28%)	7 (27%)	1.0	1 (13%)	9 (23%)	0.67
Medications						
Antiplatelet and/or anticoagulant	32 (59%)	12 (46%)	0.34	3 (38%)	24 (62%)	0.26
Antiplatelet drug use	28 (52%)	9 (35%)	0.16	2 (25%)	22 (56%)	0.14
Anticoagulant drug use	6 (11%)	2 (8%)	1.0	1 (13%)	7 (18%)	1.0
Statin use	27 (50%)	10 (38%)	0.35	4 (50%)	20 (51%)	1.0
β blocker use	14 (26%)	5 (19%)	0.59	1 (13%)	5 (13%)	1.0
Anatomic features						
Sacclurar	9 (17%)	3 (12%)	0.74	2 (25%)	7 (18%)	0.64
Comorbid common iliac artery aneurysm	28 (52%)	9 (35%)	0.16	2 (25%)	14 (36%)	0.70
Comorbid thoracic aortic aneurysm	9 (17%)	7 (27%)	0.37	1 (13%)	13 (33%)	0.40
Sac diameter at EVAR, mm	53.0 ± 6.6	53.8 ± 13.7	0.77	52.7 ± 8.9	56.8 ± 9.3	0.26
Proximal neck diameter, mm	24.2 ± 3.4	24.2 ± 4.2	0.94	22.8 ± 2.6	25.1 ± 3.6	0.051
Proximal neck length, mm	33.2 ± 9.7	29.3 ± 8.9	0.088	33.4 ± 12.4	33.0 ± 14.1	0.94
Patent inferior mesenteric artery	43 (80%)	22 (85%)	0.76	7 (88%)	30 (77%)	0.67
Inferior mesenteric artery diameter, mm	3.1 ± 0.7	3.1 ± 0.7	0.87	3.8 ± 1.0	2.9 ± 0.8	0.044
Number of patent lumbar arteries	4.3 ± 1.4	3.3 ± 1.8	0.013	5.1 ± 1.2	3.2 ± 1.6	0.0028
Postoperative details						
Stent graft volume						
Stent graft volume within 1 month after EVAR, mL	63.7 ± 14.8	62.6 ± 16.2	0.79	57.7 ± 15.7	68.3 ± 18.4	0.12
Stent graft volume 2 years after EVAR, ml	71.9 ± 18.2	73.9 ± 23.3	0.70	67.6 ± 17.0	86.9 ± 22.4	0.017
Stent graft volume ratio 2 years after EVAR	1.13 ± 0.08	1.18 ± 0.11	0.043	1.18 ± 0.10	1.28 ± 0.12	0.032
Stent graft volume ratio 2 years after EVAR ≤1.21	46 (85%)	16 (62%)	0.024	–		
Stent graft volume ratio 2 years after EVAR ≤1.22	–			6 (75%)	11 (28%)	0.019

Data are presented as number (%) or mean ± standard deviation.

EVAR: endovascular aneurysm repair

**Table 4:** Multivariate analysis of Type II endoleaks 2 years after EVAR (logistic regression model) (expanded polytetrafluoroethylene and polyester stent grafts)

Expanded polytetrafluoroethylene stent grafts (n = 80)		
Variable	OR (95% CI)	*p*-value
Age ≥80 years	3.90 (1.07–14.16)	0.039
Number of patent lumbar arteries ≥4	2.17 (0.73–6.47)	0.16
Stent graft volume ratio ≤1.21	3.73 (1.12–12.41)	0.032
Polyester stent grafts (n = 47)		
Variable	OR (95% CI)	*p*-value
Inferior mesenteric artery diameter ≥3.1 mm	15.98 (0.99–256.29)	0.051
Number of patent lumbar arteries ≥4	20.95 (1.20–366.92)	0.037
Stent graft volume ratio ≤1.22	19.31 (1.55–241.18)	0.022

OR: odds ratio; CI: confidence interval; EVAR: endovascular aneurysm repair

#### Polyester stent graft subgroup

Univariate analysis revealed that the diameter of the inferior mesenteric artery, the number of patent lumbar arteries, and the SgV ratio were significantly correlated with T2EL (**Table 3**). An SgV ratio value of 1.22 was calculated as the cut-off (positive predictive value 35%, negative predictive value 93%, and sensitivity 72%, specificity 75%, AUC 0.74). Regarding the number of patent lumbar arteries, the cut-off was calculated as 4 [sensitivity 79%, specificity 75%, AUC 0.82]. Regarding the diameter of the inferior mesenteric artery, 3.1 mm was calculated as the cut-off [sensitivity 67%, specificity 88%, AUC 0.76]. Multivariate analysis revealed that patent lumbar arteries ≥4 and SgV ratio ≤1.22 were independently associated with T2EL (**Table 4**).

## Discussion

We demonstrated that the SgV ratio is significantly associated with T2EL and is an independent factor for T2EL. This result suggests that the SgV ratio is a promising indicator of T2EL after EVAR, with a small SgV ratio implying the existence of T2EL. There have been several studies on the usefulness of aneurysm sac volumetry after EVAR,^8,9)^ but stent graft volumetry has not been studied to date. To evaluate stent graft enlargement objectively, we adopted SgV instead of stent graft diameter. The integrated value by tracing the stent graft fabric border is the true value of SgV. However, an integrated value by tracing the stent border can be considered an approximation of the true value of SgV in the stent graft that had an outer metallic skeleton. As SgV varied widely among patients, we calculated the change in SgV relative to the initial assessment after EVAR. SgL had small changes, and the correlation between SgL and the SgV ratio was low. Compared with CT findings on axial imaging, the diameter of stents increased over time. Therefore, the stent graft volume may increase because of the dilatation of the stent graft in diameter, rather than the elongation of the stent graft in length. The SgV increased 2 years postEVAR, but only compared with immediately after EVAR, and did not exceed its original size. The stent graft may not have fully expanded within 1 month postEVAR and attained its intended size over time. However, intra-aneurysmal sac pressure may suppress full expansion to the original maximum size; therefore, volume increases can differ. There are marked differences in graft behavior after EVAR between ePTFE and polyester grafts. ePTFE grafts generally show less dilatation than polyester grafts after implantation^10)^ and tend to have T2EL more frequently than polyester stent grafts.^11)^ However, even after stratification by the graft material, the SgV ratio was an independent factor in ePTFE and polyester stent grafts. Regardless of the stent graft material, a low SgV ratio may infer the existence of T2EL after EVAR. We are considering an investigation of SgV on each stent graft model eventually.

The frequency of T2EL in this study was 49%, slightly higher than the range of 20–40%.^1)^ The higher frequency of T2EL in this study may be attributed to the following: (1) the proportion of ePTFE stent grafts was high; (2) embolization of the inferior mesenteric artery or lumbar artery concomitant with EVAR was not performed routinely; and (3) some patients without T2EL who showed obvious sac shrinkage were not included because they did not undergo CT 2 years after EVAR. It is necessary to regularly check for the presence of T2EL because T2EL may disappear or emerge during follow-up.^2,5,6)^ However, regular inspections for T2EL using contrast media are problematic because contrast radiography has some disadvantages. Because SgV can be calculated without contrast media, the SgV ratio should be useful for regular estimation of T2EL. Plain CT scans, which are often used only to check the aneurysm sac size, may also be used.

Aneurysm sac pressure has been reported to be useful in detecting intraoperative Type I and III endoleaks,^12)^ and intra-aneurysm sac pressure measurement is crucial for post-EVAR surveillance, allowing early detection of exclusion failures.^13)^ After open surgical repair for AAA, the implanted graft is free from the surrounding pressure because the aneurysm sac has been opened and dilates to its own maximum size of the graft.^14–17)^ However, a stent graft is inserted into the closed space of the aneurysm sac and continues to receive intra-aneurysmal sac pressure postEVAR. The SgV ratio may reflect the intra-aneurysm sac pressure. High intra-aneurysmal sac pressure due to T2EL may suppress stent graft enlargement to its original maximum size, whereas depressurization resulting from the absence of T2EL may allow the stent graft to enlarge to nearly its original maximum size. Dias et al. reported that the mean intra-sac pressure of 6 patients who had T2EL was 19–122 mmHg and the ratio to the aortic pressure was 22%–92%.^13)^ The intra-sac pressure due to T2EL can be high enough to suppress the expansion of the stent graft after EVAR in some cases. Several types of aneurysm sac pressure measurements have been reported, but they require invasive procedures.^18)^ If the SgV ratio proves to be strongly associated with intra-aneurysm sac pressure, it may be useful as a less invasive indicator of intra-aneurysmal sac pressure.

This study had several limitations. First, it was a retrospective study. Moreover, there was selection bias, and time and effort are required to measure SgV manually. The average measurement time of an SgV was approximately 10 min. An optimal cut-off value for the SgV ratio has not been established for ePTFE and polyester stent grafts. This study suggests that the SgV ratio is correlated with T2EL, but the SgV ratio has not been proven to be directly associated with late adverse events, including reintervention, rupture, and mortality. As previously reported, in this study, the aneurysm sac diameter was also larger in patients with T2EL than in those without it, 2 years after EVAR. An association between the SgV ratio and late adverse events also needs to be verified.

However, this study suggests some significant advantages of measuring SgV. SgV based on plain CT can be helpful in T2EL surveillance. The SgV ratio may be a dynamic indicator of intra-aneurysm sac pressure. Measurement accuracy and efficiency are expected to improve with the utilization of software, enabling automated sac volume measurement.^8,9)^ We believe that the SgV ratio may improve T2EL surveillance, and further research is required to clarify the utility of the SgV ratio after EVAR.

## Conclusion

This study suggests that the SgV ratio is significantly correlated with T2EL. The SgV ratio may improve T2EL surveillance after EVAR.
